# A Wonderful Flower

**Published:** 1890-04

**Authors:** 


### A WONDERFUL FLOWER.

*Les Mondes*, a French publication, gives an account of a newly discovered flower called the snow flower. It is said to have been discovered by Count Anthoskoff in the most northern portion of Siberia, where the ground is continually covered with frost. This wonderful object shoots forth from the frozen soil only on the first of each succeeding year. It shines for but a single day, and then resolves to its original elements. The leaves are three in number, and each about three inches in diameter. They are developed only on that side of the stem toward the north, and each seems covered with microscopic crystals of snow. The flower when it opens is star shaped, its petals of the same length as the leaves, and about half an inch in width. On the third day the extremities of the anthers, which are five in number, show minute glistening specks like diamonds; about the size of a pin's head, which are seeds of this wonderful flower. Anthoskoff collected some of these seeds and carried them with him to St. Petersburg. They were placed in a pot of snow, where they remained for some time. On the first of the following January the miraculous snow flower burst through its icy covering, and displayed its beauties to the wondering Russian Royalty.

Food Value of an Egg.—Prof. Fresenius, of Wiesbaden, after a long series of chemical analyses, declares that an egg contains as much nourishment as a pound and an ounce of cherries, a pound and a quarter of grapes, a pound and a half of russet apples, two pounds of gooseberries, and four pounds of pears; and that 114 pounds of grapes, 127 pounds of russet apples, 192 pounds of pears, and 327 pounds of plums, are equal in nourishment to 100 pounds of potatoes.

